# Miniature optoelectronic compound eye camera

**DOI:** 10.1038/s41467-022-33072-8

**Published:** 2022-09-26

**Authors:** Zhi-Yong Hu, Yong-Lai Zhang, Chong Pan, Jian-Yu Dou, Zhen-Ze Li, Zhen-Nan Tian, Jiang-Wei Mao, Qi-Dai Chen, Hong-Bo Sun

**Affiliations:** 1 State Key Laboratory of Integrated Optoelectronics, College of Electronic Science and Engineering, Jilin University, 2699 Qianjin Street, 130012 Changchun, China; 2 Fluid Mechanics Key Laboratory of Ministry of Education, Institute of Fluid Mechanics, Beihang University, 100191 Beijing, China; 3 State Key Laboratory of Precision Measurement Technology and Instruments, Department of Precision Instrument, Tsinghua University, Haidian District, 100084 Beijing, China

**Keywords:** Imaging and sensing, Micro-optics

## Abstract

Inspired by insect compound eyes (CEs) that feature unique optical schemes for imaging, there has recently been growing interest in developing optoelectronic CE cameras with comparable size and functions. However, considering the mismatch between the complex 3D configuration of CEs and the planar nature of available imaging sensors, it is currently challenging to reach this end. Here, we report a paradigm in miniature optoelectronic integrated CE camera by manufacturing polymer CEs with 19~160 logarithmic profile ommatidia via femtosecond laser two-photon polymerization. In contrast to μ-CEs with spherical ommatidia that suffer from defocusing problems, the as-obtained μ-CEs with logarithmic ommatidia permit direct integration with a commercial CMOS detector, because the depth-of-field and focus range of all the logarithmic ommatidia are significantly increased. The optoelectronic integrated μ-CE camera enables large field-of-view imaging (90°), spatial position identification and sensitive trajectory monitoring of moving targets. Moreover, the miniature μ-CE camera can be integrated with a microfluidic chip and serves as an on-chip camera for real-time microorganisms monitoring. The insect-scale optoelectronic μ-CE camera provides a practical route for integrating well-developed planar imaging sensors with complex micro-optics elements, holding great promise for cutting-edge applications in endoscopy and robot vision.

## Abstract (web summary)

The defocusing problem has been considered the main bottleneck for developing optoelectronic μ-compound eye (CE) cameras. Here, the authors report miniature optoelectronic CE cameras with an ommatidia logarithmic-profile. The camera enables large field-of-view imaging, spatial position identification, and sensitive trajectory monitoring of moving targets.

## Introduction

Natural compound eyes (CEs) of insects are advanced and complex imaging systems that consist of numerous closely packed and hemispherically distributed ommatidia. Each ommatidium works as an independent photosensitive unit and cooperates with each other as a whole to realize prey recognition and enemy defense. Notably, insect CEs feature small size, distortion-free imaging, wide field-of-view (FOV), and sensitive motion tracking ability^1–4^, which inspires the innovation of artificial CEs, aiming to overcome the limitations of existing imaging technologies and promote their performance in medical endoscopy, panoramic imaging, micro navigation and robot vision^5–11^.

In the past decade, great efforts have been devoted to the development of artificial CEs through bionic manufacturing. Planar CEs cameras were first implemented by integrating a microlens array (MLA) with commercial CCD/CMOS detectors, by which high-resolution imaging is realized, whereas the FOV is limited due to the planar structure^12^. To achieve large FOV, three-dimensional (3D) CEs with insect-like structures have been successfully prepared with the help of advanced micro-nano fabrication technologies. For instance, Lee et al. prepared a bionic CE that is anatomically as well as functionally close to natural CEs by integrating curved MLAs, polymer cones, and waveguide cores fabricated via microlens templating, reconfigurable microtemplating, and self-writing in a photoresist, respectively^13,14^. Besides, 3D miniature CEs with hundreds of ommatidia have also been fabricated via femtosecond laser additive/subtractive manufacturing^15,16^. However, these CEs can only functionalize as a unique 3D MLA, their imaging performance is usually evaluated with the image acquisition system of a microscope by continuously tuning the image distance of ommatidia at different positions. The lack of integrated photodetectors significantly limits their portability and real-time monitoring ability.

To develop an optoelectronic integrated CE system, independent ommatidium (microlens) can be attached to individual photodetectors. The as-formed camera array with a curved surface distribution can work together and functionalize as an optoelectronic CE. Typically, Floreano et al. prepared an artificial CE camera (2.2 cm^3^ and 1.75 g) by cutting and assembling hard microlens and photodetectors, achieving an enhanced FOV in a single direction^17^. Rogers et al. combined a flexible lens array with a deformable hemispherical silicon photodiode array, forming a CE camera (14.72 mm in size) with a FOV as high as 140–180° ^2,3^. Since each ommatidium only contributes a pixel for imaging, their imaging resolution is relatively low, and the total size is much larger than that of an insect CE. The above-mentioned pioneer works lay a solid foundation for developing advanced CE cameras. Nevertheless, further miniaturization of the whole CE system becomes challenging due to the incompatibility of complex CE and available imaging sensors. At present, reports on optoelectronic integrated CE (μ-CE) cameras with a feature size comparable to insect ones are still rare.

In this paper, we report a bionic μ-CE camera with an integrated optoelectronic system that enables large-FOV imaging and real-time 3D monitoring of microorganisms. To overcome the defocusing problem, we fabricate a polymer CE of special surface profiles with a feature size similar to mosquito CEs via femtosecond laser two-photon polymerization (FL-TPP). Especially, the profile of each ommatidium is designed following a logarithmic function. In this way, the depth-of-filed and focus range of ommatidia can be significantly increased; and the as-obtained μ-CE can be suitably integrated with a commercial CMOS detector (OV9734), forming an optoelectronic integrated μ-CE camera.

## Results

### Design principle of an optoelectronic μ-CE camera

**Fig. 1:**
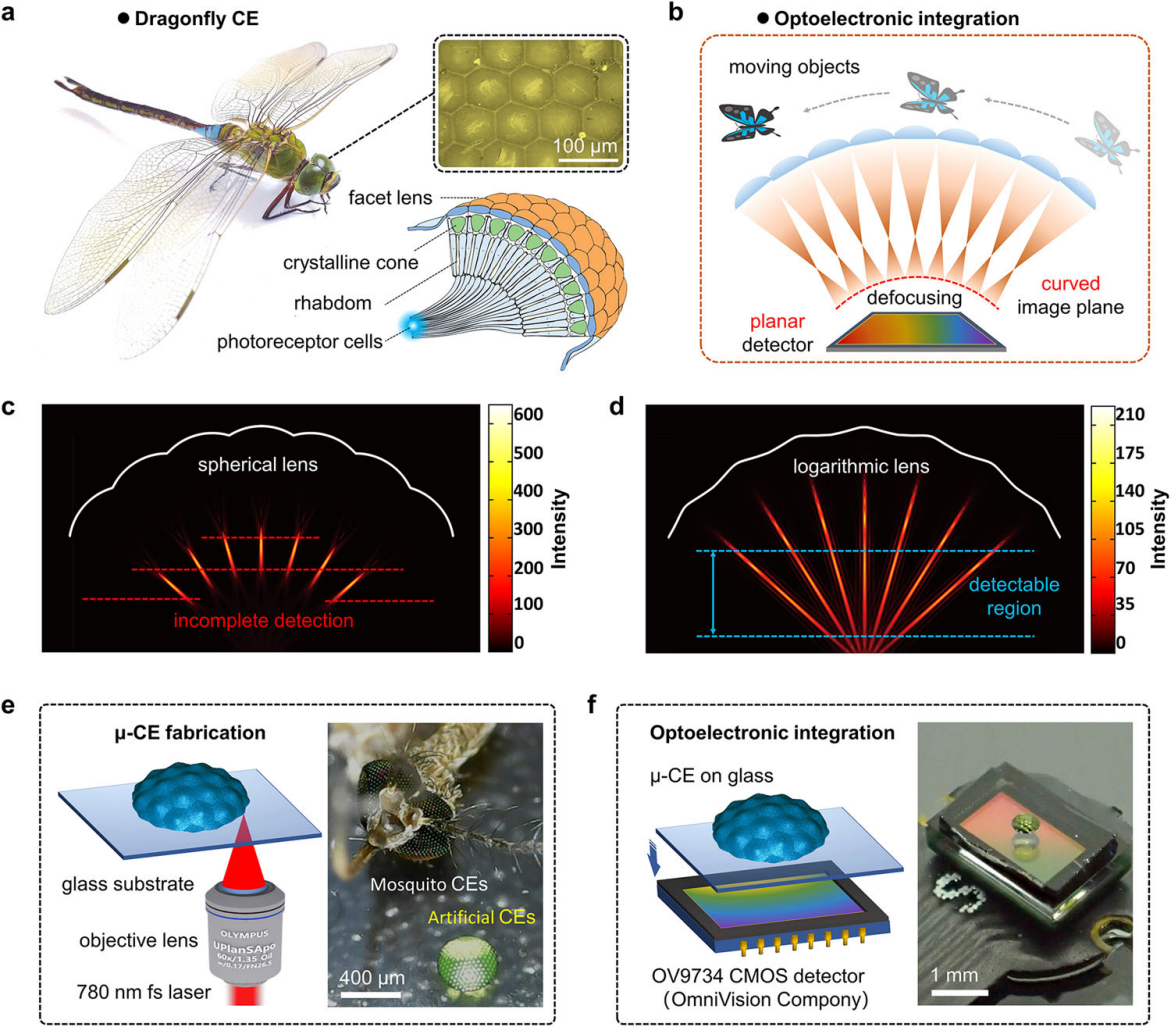
Design and fabrication of the optoelectronic CE camera. **a** Photograph of a dragonfly; the insets are the microscopic image of its CE and the schematic diagram of the organism underneath. **b** Schematic illustration of the defocusing issue in optoelectronic integration; there is a mismatch between the curved image surface and the planar image sensor. **c**, **d** Simulated light field of CEs with spherical ommatidia (**c**) and logarithmic ommatidia (**d**) via COMSOL Multiphysics simulation software; The incident light of the ommatidium was set as a plane wave. **e** The schematic diagram for the fabrication of CEs using FL-TPP; the inset is a photograph of an as-prepared μ-CE and the head of a mosquito. **f** The schematic diagram for the optoelectronic integration; the inset is the photograph of an optoelectronic integrated μ-CE camera.

Natural CE systems provide the inspiration for developing advanced imaging technologies. For instance, the CEs of a dragonfly consist of thousands of sophisticated, closely packed, and 3D distributed ommatidia that consist of facet lenses, crystalline cones, rhabdoms, and photoreceptor neural networks underneath (Fig. 1a). The cooperation of ommatidia with different orientations enables large-FOV and distortion-free imaging, as well as sensitive prey/enemy detection. However, in the case of artificial CE cameras, we have to combine micro-optic elements of complex 3D configurations (e.g., MLA distributed on a hemispherical dome) with digital photodetectors. Especially, when the overall size of CEs is close to that of insect ones, all ommatidia need to share a single image sensor. In this case, the integration of optical and electrical components becomes very tricky, since the planar detector cannot match the curved image plane. Consequently, photodetectors cannot receive all images formed by the ommatidia, which results in defocusing effect (Fig. 1b). The mismatch between the curved image plane and the planar photodetector consists of the main bottleneck for developing optoelectronic μ-CE cameras. In this regard, it is crucial to design the optical and electrical components as a whole and make the CEs more suitable for commercial planar detectors.

For microlens, the surface profile is the decisive factor that governs its optical characteristics. Nevertheless, to the best of our knowledge, most of the ommatidia of CEs resort to a simple spherical profile. Generally, to correct the optical aberrations (including spherical aberration, field curvature, coma, astigmatism, and distortion) of traditional spherical lenses, various aspheric lenses (paraboloid, hyperboloid, conical, and free-form surfaces) have been designed and prepared. Furthermore, to improve the correction effect of off-axis optical aberrations and achieve uniform imaging with a large FOV, aspheric-based multi-lens systems, and multi-aperture imaging systems are ideal solutions^18–22^. The logarithmic lens, a special aspherical lens, can produce a focal line with uniform intensity distribution, thereby effectively expanding the focus range and depth of field^23^. In this work, we design the μ-CE using logarithmic lenses as ommatidia instead of traditional spherical lenses. To make a direct comparison between CEs with spherical ommatidia and with logarithmic ones, we simulated the focused light fields of the two CEs, as shown in Fig. 1c and d. Notably, the spherical CE forms a curved focal array that can hardly match a planar detector unless ommatidia are precisely designed with different focal lengths^24^. In that case, the design and fabrication difficulties would increase sharply with the number of ommatidia, and the image size from different ommatidia varies obviously. By comparison, logarithmic CE with uniform ommatidia shows a significantly elongated focus range at the cost of energy dispersion in the focal spot, enabling planar detection.

Nevertheless, the use of aspheric microlenses as ommatidia significantly increases the fabrication difficulties of μ-CEs. Conventional technologies capable of μ-CE fabrication, for instance, photoresist thermal reflow^25^, inkjet printing^26^, laser processing assisted wet etching and thermal embossing^15,16^, cannot get precise control over the surface profiles. To address this issue, FL-TPP^27–34^, which enables arbitrary 3D fabrication, is employed to produce μ-CEs with ommatidia of function-defined surface profiles. For instance, we have previously reported the μ-CE with aspheric lens ommatidia to reduce spherical aberration in high-quality imaging^35^. Nevertheless, the resultant μ-CE is still incompatible with planar CCD/CMOS detectors due to the defocusing problem. The feature size of the as-obtained μ-CE is comparable to that of a mosquito (Fig. 1e). Interestingly, the μ-CE can be directly integrated with a commercial CMOS detector (OV9734, OmniVision Company), working as an optoelectronic integrated μ-CE camera (Fig. 1f).

### Comparisons between spherical and logarithmic CEs

To make a comprehensive comparison between spherical and logarithmic ommatidia, we first fabricated a single spherical microlens and a logarithmic microlens via two-photon polymerization using a negative tone photoresist SZ2080 and evaluated their focusing properties. The surface profile and function of the spherical ommatidium can be described as:

1 $${H}_{{SL}}\left(r,\, \theta \right)=\sqrt{{R}^{2}-{r}^{2}}$$

2 $${f}_{{SL}}=1/\left[\left({n}_{L}-{n}_{0}\right)\times \frac{1}{R}\right]$$

**Fig. 2:**
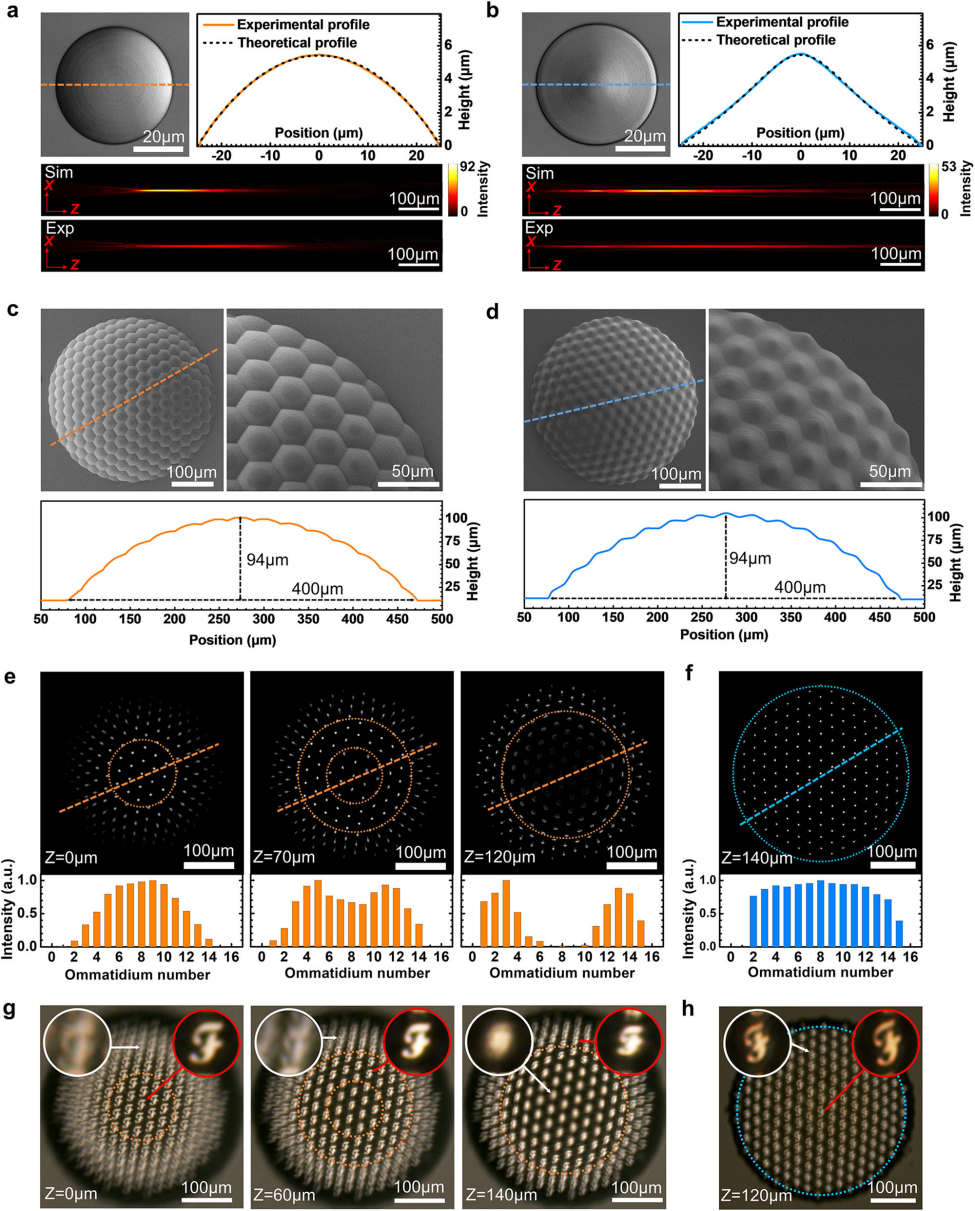
Comparisons between spherical and logarithmic CEs. **a**, **b** Optical properties of **a** a single spherical lens (SL, radius: 25 μm, focal length: 355 μm) and **b** a logarithmic lens (LL, radius: 25 μm, focusing range: 100–800 μm). The top-left images are microscopic images; the top-right results are cross-sectional profiles; the bottom images are simulative (Sim) and experimental (Exp) focusing intensity distributions along the optical axis. **c**, **d** Morphologies of the as-prepared spherical (**c**) and logarithmic (**d**) μ-CEs; top images are overall and magnified SEM images, and the bottom results are relative cross-section profiles. **e**, **f** Focusing images (top) and normalized intensity distributions along the dotted line (bottom) of spherical (**e**) and logarithmic (**f**) μ-CEs. **g**, **h** Imaging results of spherical (**g**) and logarithmic (**h**) μ-CEs; the insets are magnified images of different ommatidia.

And the surface profile function of the logarithmic lens can be described as:

3 $$a=\frac{{d}_{2}-{d}_{1}}{{\left(\frac{D}{2}\right)}^{2}}$$

4 $${\varphi }_{{LL}}\left(r,\, \theta \right)=-\frac{1}{2a}{{{{{{\mathrm{ln}}}}}}}\left\{\left.2a{\left[{a}^{2}{r}^{4}+\left(1+2a{d}_{1}\right){r}^{2}+{d}_{1}^{2}\right]}^{\frac{1}{2}}+2{a}^{2}{r}^{2}+1+2a{d}_{1}\right\}\right.*\frac{2\pi}{ \lambda }$$

5 $${H}_{{LL}}\left(r,\, \theta \right)=\frac{{\varphi }_{{LL}}\left(r,\, \theta \right)}{{n}_{L}-{n}_{0}}*\frac{\lambda }{2\pi }$$

where *r is* radius coordinates, *θ*, azimuth coordinates, *D*, lens diameter, *n*_L_ and *n*_0_, lens refractive index, and environmental refractive index, respectively. *R*, curvature radius, *f*_SL_, the focal length of the spherical lens, *d*_1_ and *d*_2_, and the start and end points of the logarithmic lens’s focal lines, respectively. To compare the focusing range and depth-of-field of these two lenses, a spherical ommatidium (*D* = 50 μm, *f*_SL_ = 355 μm) and a logarithmic ommatidium (*D* = 50 μm, *d*_1_ = 100 μm, *d*_2_ = 800 μm) were prepared (Fig. 2a, b). With reasonably high 3D processing precision, FL-TPP can guarantee the high fidelity of their profiles, in which the experimental profiles match well with the theoretical functions. In the focusing tests, a continuous-wave He-Ne laser with a wavelength of 632.8 nm was used for focusing and imaging tests in water. Notably, both the theoretical simulations and experimental results confirm that the logarithmic lens shows a much larger focus range and depth-of-field than that of the spherical lens. The focus ranges of the spherical lens and the logarithmic one are 360 and 705 μm, respectively. Correspondingly, the depth-of-field of the spherical lens and the logarithmic ones is 200 and 440 μm, respectively, (Supplementary Fig. 1).

The unique focusing properties of the logarithmic lens make it possible to solve the defocusing problem of CEs with spherical ommatidia. To provide solid evidence, we fabricated a spherical CE (control experiment) and a logarithmic one (Fig. 2c, d), respectively. These two CEs were designed by closely arranging ~160 ommatidia of uniform size (spherical ommatidium: *D* = 40 μm, *f*_SL_ = 240 μm; logarithmic ommatidium: *D* = 40 μm, *d*_1_ = 50 μm, *d*_2_ = 600 μm) on a spherical lens dome (400 μm in diameter and 90 μm in height). In this way, the fill factor can reach 100%, which can provide a higher light utilization rate and signal-to-noise ratio. Scanning electron microscope (SEM) images confirm the high surface smoothness and the distinct profiles of these two CEs. The cross-section profiles extracted from the laser confocal microscope (LSCM, Supplementary Fig. 2) along the dotted lines provide more details with respect to the size of the CEs and the profile of their ommatidia. Besides, the statistics on the uniformity of each ommatidium on these two CEs are also provided (Standard deviations for the diameter of spherical and logarithmic ommatidia are 0.62 and 0.57 μm, respectively. And standard deviations for the height of spherical and logarithmic ommatidia are 0.16 and 0.15 μm, respectively, Supplementary Fig. 3), indicating the uniformity of ommatidia.

To compare their imaging performance, we first investigated their focusing properties using a microscopic imaging system with a ×10/0.25NA objective lens (Supplementary Fig. 4). By tuning the image distance, the focusing region varies from the center to the outer region (Fig. 2e, see the dotted circle). Relative focus spot intensity distributions along the orange dotted line further confirm the change of focus region. Obviously, for spherical CE, it is impossible to detect the clear focal spots from all the ommatidia at one time, indicating the defocusing problem. In contrast, due to the enlarged focus range, all the clear focus spots can be collected at one time in the case of logarithmic CE (Fig. 2f), as confirmed by the focus spot intensity distribution. The subsequent imaging tests further confirm their different imaging abilities. Here, we employed the bright letter “F” as an object. By tuning the position of the spherical CE, the ommatidia in the focused region can form a clear image, whereas the images from the rest of the ommatidia are out-of-focus, as shown in the insets of Fig. 2g. For the logarithmic CE, clear images can be collected from all the ommatidia, simultaneously (Fig. 2h). Based on the comprehensive comparison between these two CEs, we can conclude that the spherical CE suffers from the defocusing problem, whereas the logarithmic CE is capable of integration with a planar CCD/CMOS detector, despite the image brightness is reduced due to the energy dispersion.

**Fig. 3:**
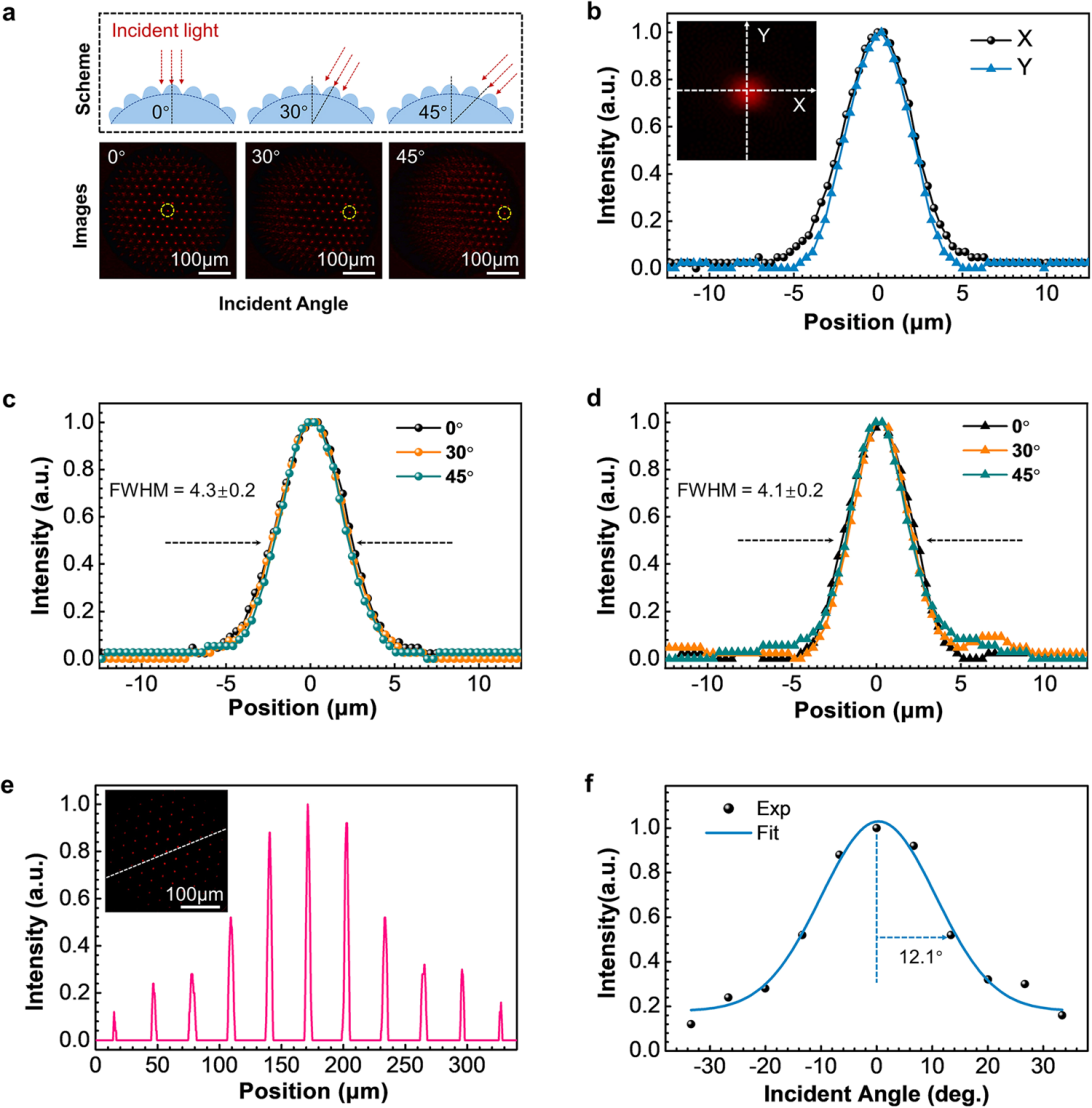
Measurement of FOV and angular sensitivity function (ASF) of the logarithmic μ-CE. **a** Schematic illustration (top) and the focused light field images (bottom) of the logarithmic μ-CE; the incident angles are 0°, 30°, and 45°, respectively. **b** Comparison of the intensity distributions along the *X*-axis and *Y*-axis at normal incidence; the insert is an image of a focal point. **c**, **d** Normalized intensity distribution along the *X*-axis (**c**) and *Y*-axis (**d**) under different incident angles (0°, 30°, and 45°); the wavelength of the continuous light is 633 nm. **e** The normalized intensity distribution under normal incidence extracted from the focusing image (inset) along the dotted line. **f** ASF of the logarithmic μ-CE (FWHM = 12.1°).

To get deep insight into the optical properties of the logarithmic CE, we also investigate its FOV and the angular sensitivity function (ASF). According to the numerical derivation, the FOV of a CE can be calculated by the following formula:

6 $${{{{{{\mathrm{FOV}}}}}}}=2{\arcsin }\left(\frac{{H}_{m}{D}_{m}}{{{H}_{m}}^{2}+{\left(\frac{{D}_{m}}{2}\right)}^{2}}\right)$$

where *H*_*m*_ and *D*_*m*_ are the height and the diameter of the main lens dome. Obviously, the FOV of CEs is governed by the geometry of the main lens dome where the ommatidia are distributed. According to the parameters of our CE model, its theoretical FOV is 96.9°. To evaluate the FOV experimentally, optical micrographs at different incident angles (0°, 30°, and 45°) were collected (Fig. 3a), in which the deflection of the focused region can be detected. And the best focus area always follows the light incident angle to move. Besides, to investigate the focusing performance, the point spread functions (PSF) of a focused spot along the *X*-axis and *Y*-axis were measured under normal incidence (Fig. 3b). The focused spot appears as a standard circle, and its intensity is Gaussian, indicating that the ommatidium has a high processing quality that meets the design. Normalized intensity distributions along the *X*-axis and *Y*-axis under different incident angles are plotted in Fig. 3c, d, respectively. The full width at half maximum (FWHM) almost remains constant (*X*-axis: FWHM = 4.3 ± 0.2, *Y*-axis: FWHM = 4.1 ± 0.2), indicating the low aberration in a wide FOV. The slight difference in FWHM along the two directions can be attributed to the non-absolute normal incidence. In addition to FOV, ASF, which represents the sensitivity to moving objects, is another key parameter of CEs. Under normal incidence, normalized intensity distributions of the ommatidia along the dotted line were extracted from the optical image (Fig. 3e), and the intensity distribution was plotted as a function of the incidence angle and Gaussian fitting (Fig. 3f). The measured FWHM value of the ASF was 12.1°. As a control experiment, the FOV and ASF of the spherical CE are 90° and 19.3°, respectively (Supplementary Fig. 5). The two CEs show similar FOV due to the same parameters of the dome. In addition, the logarithmic CE demonstrates higher angular sensitivity due to the faster attenuation of the focal intensity at oblique incidence. It is worth pointing out that, with designable 3D processing ability, FL-TPP enables the direct fabrication of CEs with arbitrary 3D geometries. Consequently, the FOV can be tuned by varying the size of the dome. Nevertheless, to integrate a single lens CE with a planar CMOS detector, a suitable FOV is ~90°. From the viewpoint of TPP fabrication, it is undoubtedly possible to fabricate compound eyes with much larger FOV. Nevertheless, considering the imaging quality and the utilization of ommatidia at the marginal part, it is not necessary to pursue a larger FOV. Through the comprehensive comparison, it can be concluded that the logarithmic CE shows similar optical performance to the spherical one, while the former can well address the defocusing problem.

### Optoelectronic integrated CE camera

**Fig. 4:**
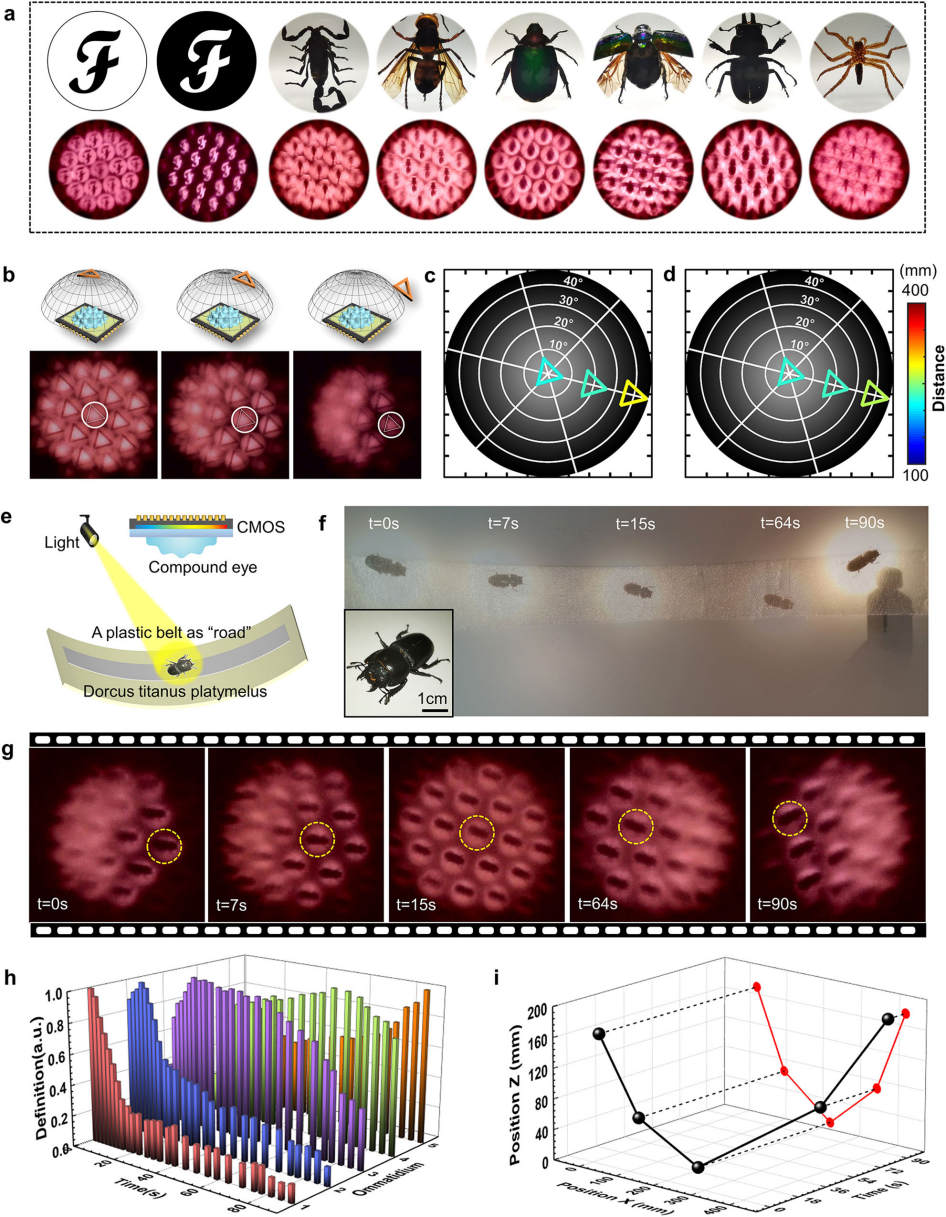
The imaging capability of the optoelectronic μ-CE camera. **a** Photographs of different targets (top) and the images collected by the μ-CE camera (bottom). **b** Schematic illustrations of the different spatial positions of a triangle model (top) and relative images captured by the μ-CE camera (bottom). **c** The true spatial position of the triangle relative to the μ-CE camera. **d** The calculated spatial position of the triangle according to the images; the side length of the triangular is known (20 mm). The radius of the imaging FOV is 80 mm. **e** Schematic diagram of the experimental setup for monitoring beetle motion using the μ-CE camera. **f** Time-lapse photography of a free-crawling beetle at different moments. The photograph is generated by combining five photographs together. The inset is the photograph of the beetle. **g** Images captured by the μ-CE camera at different times. **h** The definition statistics of five ommatidia (marked in circles) extracted from the images captured by the μ-CE camera at different times. The definition of ommatidia in different positions reaches the maximum at different moments. **i** The calculated spatial positions of the beetle at different moments and the as-generated movement trajectory.

The use of logarithmic CE makes it possible to integrate the CE with commercial CMOS detectors. In this work, to improve imaging quality, we optimized the parameters of ommatidia (*D* = 110 μm, *d*_1_ = 500 μm, *d*_2_ = 1000 μm) and keeps the dome unchanged (400 μm in diameter and 90 μm in height). A designed 19-ommatidia CE was fabricated as the optical component and combined with a CMOS photosensitive chip (Omnivision 9734, 2.5 × 1.7 mm) as electrical components, achieving optoelectronic integration (Supplementary Figs. 6 and 7). The CE with a feature size of ~400 μm can cover more than 80,000 pixels which can guarantee a reasonable imaging resolution. The optoelectronic CE camera enables directly capturing images of different types of target objects. As shown in Fig. 4a, clear images including bright-field/dark-field “F” and various insects can be formed by every ommatidium (Magnified images are shown in Supplementary Fig. 8, high background noise may originate from light spillage under oblique incidence and light incidence in the blank area outside the lens. This problem can be well solved by adding baffles, but for TPP, it is challenging).

As a μ-CE camera, the cooperation of ommatidia enables sensitive trajectory monitoring of moving targets. It is well known that insect CE vision is very sensitive to moving objects. When the target moves, the ommatidia of the CE image, in turn, produce a flicker effect. In this way, insects can perceive the moving trajectory and speed of their predators or preys in real-time, and make effective feedback immediately. Inspired by insect CEs, this optoelectronic μ-CE camera is expected to achieve similar functionality. Briefly, the relative direction and distance of the target can be determined by the image definition of different ommatidia and the image size, respectively. To establish the relationship between image size and object distance, we calibrated the object-image relationship of the μ-CE camera first (Supplementary Figs. 9 and 10). To verify its position identification ability, a triangular object of known size (side length: 20 mm) was placed in three spatial positions, and corresponding images were captured by the μ-CE camera (Fig. 4b). The real spatial positions of the triangle (the distance and azimuth) in the three cases are quantified as 220 mm/0°, 233 mm/19.3°, and 282 mm/38.8°, respectively (Fig. 4c). For comparison, the reconstructed distance and azimuth for the three cases are 226 mm/0°, 233 mm/19.4°, and 262 mm/38.8°, respectively (Fig. 4d), in good agreement with the real values.

To assess its application in moving trajectory reconstruction, we recorded the motion of a living beetle using this μ-CE camera. The schematic diagram of the experiment is shown in Fig. 4e (Supplementary Fig. 11 for details). Time-lapse images of the beetle at different moments are captured by a traditional digital camera (Fig. 4f) and our optoelectronic CE camera (Fig. 4g), respectively. In the video recorded by the μ-CE camera (Supplementary Movie 1), the image definition of different ommatidia at different times and positions can be calculated (Fig. 4h), in which the distance and azimuth angle of the beetle can be determined simultaneously. In this way, the spatial positions of the beetle at different times can be reconstructed (Fig. 4i). The moving trajectory reconstruction ability makes the optoelectronic μ-CE a preferred vision system for miniature robots.

### On-chip camera for living microorganisms

**Fig. 5:**
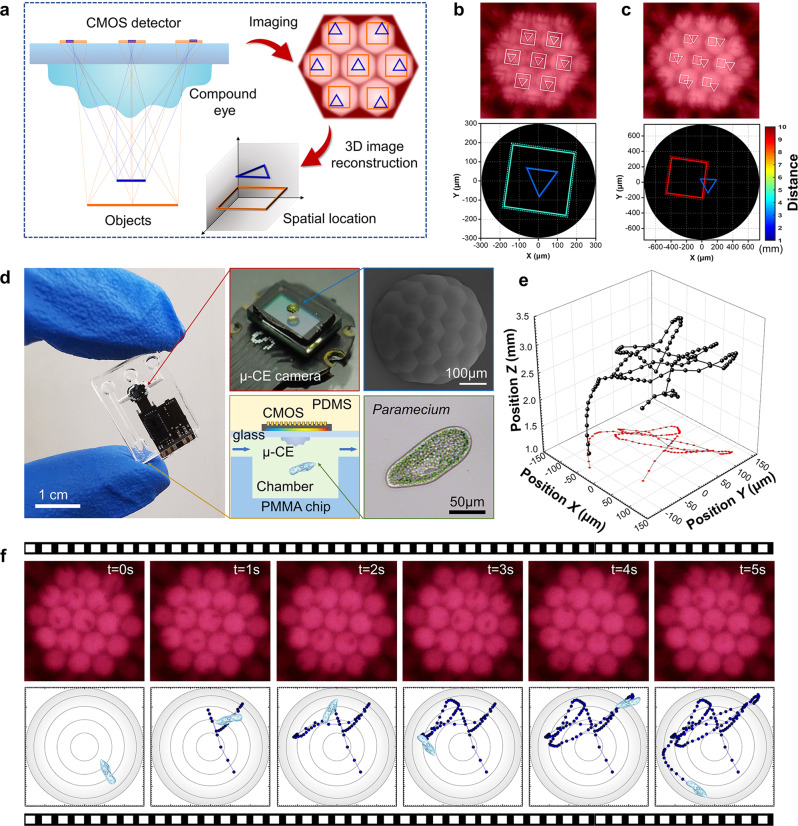
On-chip camera for living microorganisms. **a** Schematic diagram of the basic principle for microscopic 3D reconstruction based on a μ-CE camera. **b**, **c** Images captured by the μ-CE camera (top) and the 3D reconstruction results of the target objects (a micro-square and a triangle) at two different spatial positions. Dashed and solid lines represent real and reconstructed spatial positions, respectively. **d** Photograph of the on-chip camera system (left), the μ-CE camera (top-middle), and the SEM image of the logarithmic CE (top-right). The insets are schematic illustrations of its working mechanism (bottom-middle) and the microscopic image of a *Paramecium*. **e** The reconstructed 3D trajectory of the *Paramecia*. **f** Images of the *Paramecium* captured by the μ-CE camera (top) and the reconstructed spatial positions (bottom) at different moments. The radius of the imaging FOV is 150 μm.

Unlike a monocular camera that can only determine the object’s distance upon knowing its true size, the μ-CE camera enables 3D detection of the object trajectory based on the principle of multi-eye vision. When we observe the target objects using the μ-CE camera, ommatidia with different orientations can image the same target from different view angles. Notably, the as-obtained image array may be slightly different from each other in size and position deviations. By processing the instantaneous 3D imaging information of the target objects, real-time spatial location can be directly reconstructed (Fig. 5a). Here, a machine learning method was utilized to calibrate the μ-CE camera based on the back propagation (BP) neural network^36^. In the calibration of the μ-CE camera, the imaging of target objects with known parameters is firstly implemented (Supplementary Fig. 12, Due to the small focal length, the μ-CE camera has a large depth of field. Experiments have proved that clear imaging can be achieved for objects with a distance greater than 1.4 mm). After the calibration, micro squares and triangles of different sizes are placed in different spatial positions. The images captured by the μ-CE camera and the reconstructed spatial positions are presented in Fig. 5b and c, in which the dashed and solid lines represent real-case and reconstructed spatial positions, respectively. The reconstructed parameters are in good agreement with the real-case values. (Unlabeled original images, magnified 3D reconstruction results, and detailed data statistics are shown in Supplementary Fig. 13).

In addition, to demonstrate a proof-of-concept application, we proposed an advanced, miniature, and portable on-chip camera system by integrating the μ-CE camera with a microfluidic chip (Fig. 5d), which was further employed in monitoring the real-time motion trajectory of a living microorganism, *Paramecium*. The photographs of the on-chip camera and its core component (the μ-CE camera) are shown in Fig. 5d. In the real-case observation, a green living *Paramecium* with algae symbiosis was employed as a dynamic target; it is trapped in a reservoir on the microfluidic chip underneath the μ-CE camera. As a representative demonstration, a paramecium motion video was recorded by the μ-CE camera, and the real-time trajectory reconstruction is performed at a rate of 24 frames per second (Fig. 5e, Supplementary Movie 2). The time-lapse images at different moments and their instantaneous position are shown in Fig. 5f. The μ-CE camera has revealed the capability for microscopic 3D trajectory reconstruction, which is very promising for microscopic stereo imaging, microscopic flow field measurement, and real-time tracking monitoring.

## Discussion

After millions of years of evolution, natural *Arthropods* have possessed advanced visual systems, which provide intriguing inspiration for developing compact and miniature cameras. Generally, CEs consist of thousands of omnidirectionally distributed ommatidia that point in different directions and images independently, which enables wide FOV detection. Nevertheless, to achieve similar imaging capability, we have to develop artificial CE cameras that work in a completely different way from natural CEs, in which a swarm of microlenses with complex 3D arrangement has to be integrated with available photodetectors. The main challenge to reach this end is how to overcome the mismatch between nonplanar imaging with respect to omnidirectionally distributed ommatidia and planar detection with respect to commercial CCD/CMOS detectors when the feature size of the optoelectronic system decreased to micro-scale. To address the defocusing problems of 3D CEs, typical solutions, including an optical relay system^37^, multi-layer lens assembly^22,38^, curved multi-focus^24^, or the use of curved photodetectors^3,17^ have been successfully reported, which is of great significance to developing optoelectronic CE cameras (Supplementary Table 1). Nevertheless, from the practical point of view, these strategies are more or less limited in enormous difficulties with respect to fabrication, assembly, and integration. At present, the development of optoelectronic CE cameras is still at an early stage, there is a big space to make innovation on this cutting-edge topic.

In this paper, we addressed this issue by designing and producing artificial μ-CEs with logarithmic-profile ommatidia via TPP fabrication. To confirm this idea, spherical and logarithmic CEs of the same size were fabricated in the same way, and their imaging performances were compared in detail. As compared with CEs with spherical ommatidia, the defocusing problem can be effectively avoided in the case of logarithmic CEs, because the depth-of-field and focus range of all the logarithmic ommatidia are significantly increased. Inevitably, this scheme will result in a loss of energy, which darkens the image slightly. In this way, the as-obtained μ-CEs can be well integrated with a commercial CMOS detector (OV9734, Omnivision), forming an optoelectronic μ-CEs camera. Importantly, the feature size of the μ-CEs is only ~400 μm, similar to the CEs of a mosquito; and the total weight of the μ-CEs camera (including the COMS chip) is only 230 mg. With 19–160 logarithmic ommatidia that can image independently and simultaneously, our optoelectronic μ-CE camera enables large-FOV imaging (90°), spatial position identification, and sensitive trajectory monitoring of moving targets. In a typical demonstration, the moving trajectory of a living beetle can be reconstructed based on the real-time video recorded by the μ-CE camera. Furthermore, taking advantage of the small size and the unique imaging ability, the miniature μ-CE camera can be integrated with microfluidic devices, serving as an on-chip camera for real-time monitoring of living microorganisms. A machine learning method was employed to calibrate the μ-CE camera based on the back propagation (BP) neural network, in which the imaging of target objects with known parameters is first implemented. Based on the calibration, the 3D motion trajectory of a *Paramecium* in 5 s has been reconstructed from the real-time video.

In short, the development of miniature μ-CE cameras with integrated optoelectronic systems is very important, which makes it possible to see the world from the perspective of insects. Featuring small feature size, lightweight, portability, and multi-ommatidia omnidirectional imaging ability, the μ-CE camera holds great promise for cutting-edge applications in robotic vision, medical endoscopes, miniature navigation, moving target tracking, and many other micro-vision fields.

## Methods

### Photoresist preparation

An organic-inorganic hybrid photoresist SZ2080 (IESL-FORTH)^39,40^ with 1% of photosensitizer (4,4-Bis(diethylamino)benzophenone) was dropped onto a precleaned cover glass. Then, the sample was heated on a hot plate at 100 °C for 1 h to remove the organic solvent. After solidification, the sample was cooled to room temperature for use.

### Fabrication of CEs

In this experiment, a commercial galvanometer-based FL-TPP processing system (Maleon Nano system, Jicheng ultrafast equipment co. LTD) was employed for the fabrication. First, the near-infrared laser (ErFemto-780MP: central wavelength of 780 nm, a pulse length of 100 fs, pulse repetition frequency of 80 MHz) is tightly focused into the resin by a high numerical aperture objective lens (×60, NA = 1.35, Olympus). Then, 3D scanning of light spots can be realized with the help of a 2D galvanometer and a 1D piezoelectric platform. The laser power measured in front of the objective lens was 18–20 mW. The processing data were converted into a 3D point cloud with a spacing of 200 nm and the exposure time at a single point of 300 μs. By optimizing the laser processing parameters, surface roughness as low as 6 nm can be achieved, which is much lower than λ/20 (λ, the working wavelength, Supplementary Fig. 14). After processing, the sample was soaked in the *n*-propanol solution for 40 min to remove the unexposed photoresist. Besides, a DRS-TPP processing technology was used to shorten the processing time (Details can be found in Supplementary Fig. 15)^41^. Consequently, it only takes ~1.5 h to fabricate a CE lens of 400 μm. All of these processes are implemented in a yellow light environment to avoid the overall exposure of the sample. After development, the CE sample was quickly dried in the air and irradiated under a high-power ultraviolet lamp (wavelength: 365 nm, power: about 2w) for 12 h to increase the optical transmittance in the visible band (photobleaching). Long-time ultraviolet photobleaching can effectively improve the optical transmittance of the device. Finally, at a wavelength of 633 nm, more than 93% of transmittance can be achieved (Supplementary Fig. 16).

### Optoelectronic integration of the CE camera

To facilitate the packaging process, the substrate (cover glass) of the as-obtained CE was cut into a size of 1.5 × 2.0 mm^2^ using a diamond wire cutter (STX-202AQ), keeping the CE in the center. Then, CE, together with the glass substrate, was attached to a commercial miniature CMOS chip (total active array size of 1280 × 720 pixel², and each pixel has a size of 1.4 × 1.4 μm²). A UV curable adhesive NOA61 (Norland) was used to fix them (Supplementary Fig. 17).

### Structure characterization

The surface morphology of the structure is characterized by a field emission electron microscope (SEM, JSM-7500F, JEOL). The 3D profile of the sample was characterized by a laser scanning confocal microscope (LSCM, OLS4100, Olympus). Optical images of the sample were obtained using a transmission optical microscope (CX41, Olympus). The transmission spectrum of the structure was measured by a homemade micro-area transmission test system with a high-sensitive spectrometer (ULS2048x64-EVO, Avantes).

## Supplementary information

Supplementary Information

Description of Additional Supplementary Files

Supplementary Movie 1

Supplementary Movie 2

## Data Availability

The data that support the findings of this study are available from the corresponding author upon request. Source data are provided with this paper.

## Source data

Source Data

## Code availability

All the relevant code used to generate the results in this paper and Supplementary information is available upon request.

## References

[1] Brady DJ (2012). Multiscale gigapixel photography. Nature.

[2] Ko HC (2008). A hemispherical electronic eye camera based on compressible silicon optoelectronics. Nature.

[3] Song YM (2013). Digital cameras with designs inspired by the arthropod eye. Nature.

[4] Ma ZC (2019). Smart compound eyes enable tunable imaging. Adv. Funct. Mater..

[5] Lee GJ, Choi C, Kim DH, Song YM (2018). Bioinspired artificial eyes: optic components, digital cameras, and visual prostheses. Adv. Funct. Mater..

[6] Iyer, V., Najafi, A., James, J., Fuller, S. & Gollakota, S. Wireless steerable vision for live insects and insect-scale robots. *Sci. Robot*. **5**, eabb0839 (2020). 10.1126/scirobotics.abb0839 33022605

[7] Li J (2020). Ultrathin monolithic 3D printed optical coherence tomography endoscopy for preclinical and clinical use. Light Sci. Appl..

[8] Yanny K (2020). Miniscope3D: optimized single-shot miniature 3D fluorescence microscopy. Light Sci. Appl..

[9] Pahlevaninezhad H (2018). Nano-optic endoscope for high-resolution optical coherence tomography in vivo. Nat. Photonics.

[10] Lin RJ (2019). Achromatic metalens array for full-colour light-field imaging. Nat. Nanotechnol..

[11] Luo Y (2021). Varifocal metalens for optical sectioning fluorescence microscopy. Nano Lett..

[12] Tanida J (2001). Thin observation module by bound optics (TOMBO): concept and experimental verification. Appl. Opt..

[13] Lee LP, Szema R (2005). Inspirations from biological optics for advanced photonic systems. Science.

[14] Jeong K-H, Kim J, Lee LP (2006). Biologically inspired artificial compound eyes. Science.

[15] Deng Z (2016). Dragonfly‐eye‐inspired artificial compound eyes with sophisticated imaging. Adv. Funct. Mater..

[16] Liu XQ (2019). Rapid engraving of artificial compound eyes from curved sapphire substrate. Adv. Funct. Mater..

[17] Floreano D (2013). Miniature curved artificial compound eyes. PNAS.

[18] Thiele S, Arzenbacher K, Gissibl T, Giessen H, Herkommer AM (2017). 3D-printed eagle eye: Compound microlens system for foveated imaging. Sci. Adv..

[19] Gissibl, T., Thiele, S., Herkommer, A. & Giessen, H. Two-photon direct laser writing of ultracompact multi-lens objectives. *Nat. Photonics* **10**, 554–560 (2016).

[20] Hao C (2020). Single‐layer aberration‐compensated flat lens for robust wide‐angle imaging. Laser Photonics Rev..

[21] Juodkazis S (2016). 3D printed micro-optics. Nat. Photonics.

[22] Toulouse A (2022). Ultra-compact 3D-printed wide-angle cameras realized by multi-aperture freeform optical design. Opt. Express.

[23] Golub I, Chebbi B, Shaw D, Nowacki D (2010). Characterization of a refractive logarithmic axicon. Opt. Lett..

[24] Chen J (2017). Hybrid imprinting process to fabricate a multi-layer compound eye for multispectral imaging. Opt. Express.

[25] Kim K, Jang KW, Ryu JK, Jeong KH (2020). Biologically inspired ultrathin arrayed camera for high-contrast and high-resolution imaging. Light Sci. Appl..

[26] Luo Y (2013). Direct fabrication of microlens arrays with high numerical aperture by ink-jetting on nanotextured surface. Appl. Surf. Sci..

[27] Li R (2020). Stimuli-responsive actuator fabricated by dynamic asymmetric femtosecond bessel beam for in situ particle and cell manipulation. ACS Nano.

[28] Ma Z-C (2020). Femtosecond laser programmed artificial musculoskeletal systems. Nat. Commun..

[29] Juodkazis S (2020). Laser polymerized photonic wire bonds approach 1 Tbit/s data rates. Light Sci. Appl..

[30] Liu Y (2019). Structural color three-dimensional printing by shrinking photonic crystals. Nat. Commun..

[31] Ni J (2021). Gigantic vortical differential scattering as a monochromatic probe for multiscale chiral structures. PNAS.

[32] Ni J (2021). Giant helical dichroism of single chiral nanostructures with photonic orbital angular momentum. ACS Nano.

[33] Park SH, Yang DY, Lee KS (2009). Two-photon stereolithography for realizing ultraprecise three-dimensional nano/microdevices. Laser Photonics Rev..

[34] Xin, C. et al. Environmentally adaptive shape-morphing microrobots for localized cancer cell treatment. *ACS Nano* **15**, 18048–18059 (2021). 10.1021/acsnano.1c06651 34664936

[35] Wu D (2014). Bioinspired fabrication of high‐quality 3D artificial compound eyes by voxel‐modulation femtosecond laser writing for distortion‐free wide‐field‐of‐view imaging. Adv. Opt. Mater..

[36] Goi E (2021). Nanoprinted high-neuron-density optical linear perceptrons performing near-infrared inference on a CMOS chip. Light Sci. Appl..

[37] Shi C (2017). SCECam: a spherical compound eye camera for fast location and recognition of objects at a large field of view. Opt. Express.

[38] Zhang H (2013). Development of a low cost high precision three-layer 3D artificial compound eye. Opt. Express.

[39] Ovsianikov A (2008). Ultra-low shrinkage hybrid photosensitive material for two-photon polymerization microfabrication. ACS Nano.

[40] Gailevičius D (2019). Additive-manufacturing of 3D glass-ceramics down to nanoscale resolution. Nanoscale Horiz..

[41] Hu Z-Y (2021). Two-photon polymerization nanomanufacturing based on the definition–reinforcement–solidification (DRS) strategy. J. Light. Technol..

