# Supplementary material for: Miniature optoelectronic compound eye camera
Source: Nat Commun. 2022 Sep 26;13:5634. doi: 10.1038/s41467-022-33072-8 (PMC9513083; doi:10.1038/s41467-022-33072-8)
Supplement: Supplementary file 1 — Supplementary Information [file 41467_2022_33072_MOESM1_ESM.pdf]

Supplementary Information

Miniature optoelectronic compound eye camera

Zhi-Yong Hu,[1] Yong-Lai Zhang,[1,*] Chong Pan,[2] Jian-Yu Dou,[2] Zhen-Ze Li,[1] Zhen-Nan Tian,[1] Jiang-Wei Mao,[1] Qi-Dai Chen,[1] and Hong-Bo Sun[1,3]

[1] State Key Laboratory of Integrated Optoelectronics, College of Electronic Science and Engineering, Jilin University, 2699 Qianjin Street, Changchun 130012, China.

[2] Fluid Mechanics Key Laboratory of Ministry of Education, Institute of Fluid Mechanics, Beihang University, Beijing 100191, China.

[3] State Key Laboratory of Precision Measurement Technology and Instruments, Department of Precision Instrument, Tsinghua University, Haidian District, Beijing 100084, China.

Email: yonglaizhang@jlu.edu.cn

Keywords: compound eye camera, optoelectronic integration, femtosecond laser two-photon polymerization, wide field-of-view imaging, on-chip camera

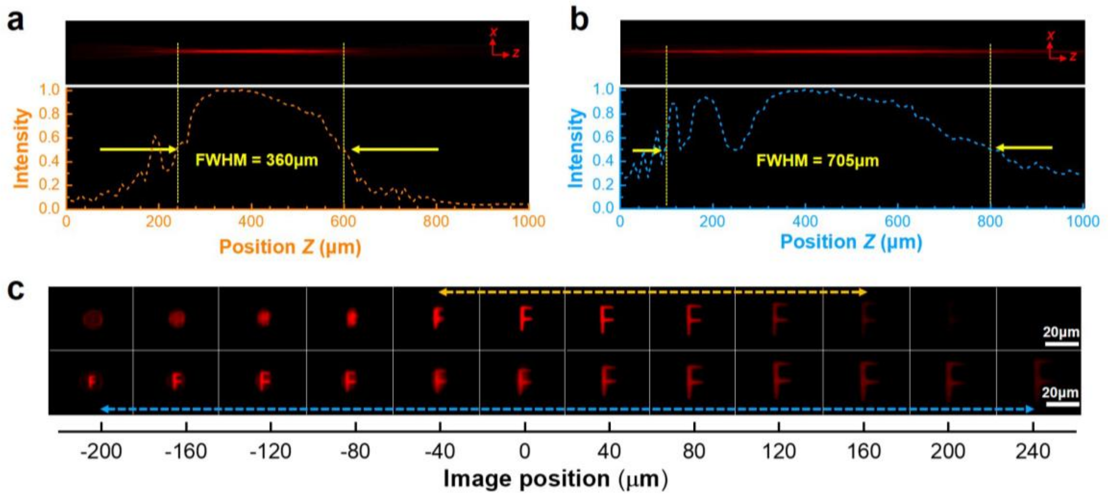

Supplementary Figure 1 | (a, b) Intensity distribution of focused spots of (a) the spherical lens and (b) the logarithmic lens along the optical axis (Experimental results). (c) Depth of field tests: the imaging of the letter "F" at different positions on the optical axis based on the spherical lens (top) and the logarithmic one (bottom). Color arrows represent the clear imaging ranges.

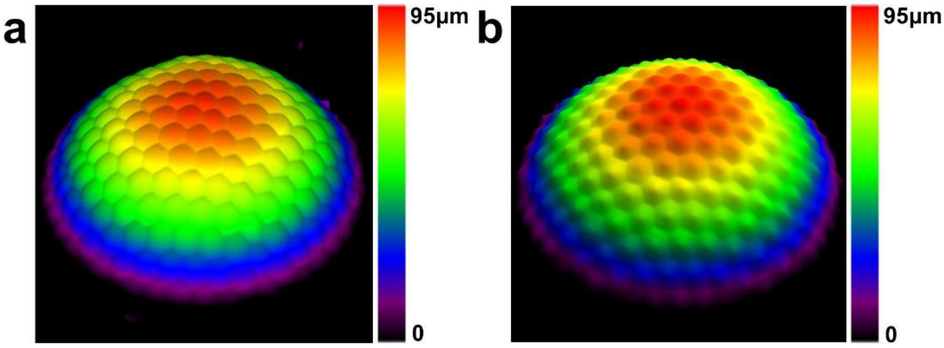

Supplementary Figure 2 | 3D LSCM images of fabricated μ-CEs. (a) Spherical μ-CE. **(b)** Logarithmic μ-CE.

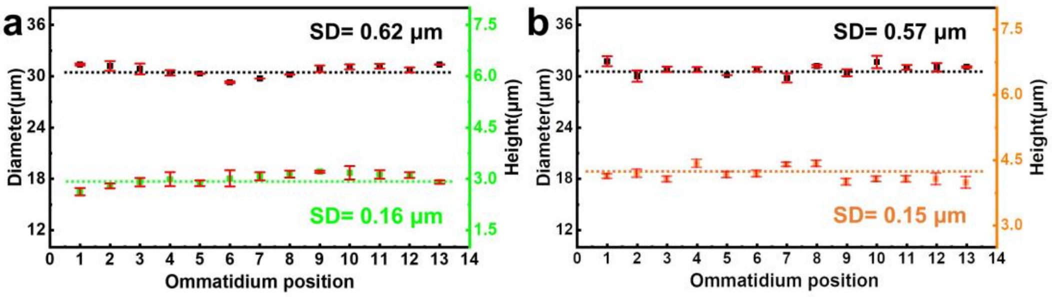

Supplementary Figure 3 | Height and diameter statistics of ommatidia from the prepared μ-CEs. (a) Spherical μ-CE: Standard deviation of diameter and height are 0.62 μm and 0.16 μm, respectively. **(b)** Logarithmic μ-CE: Standard deviation of diameter and height are 0.57 μm and 0.15 μm, respectively. Error bars denote the standard deviation of the measurements.

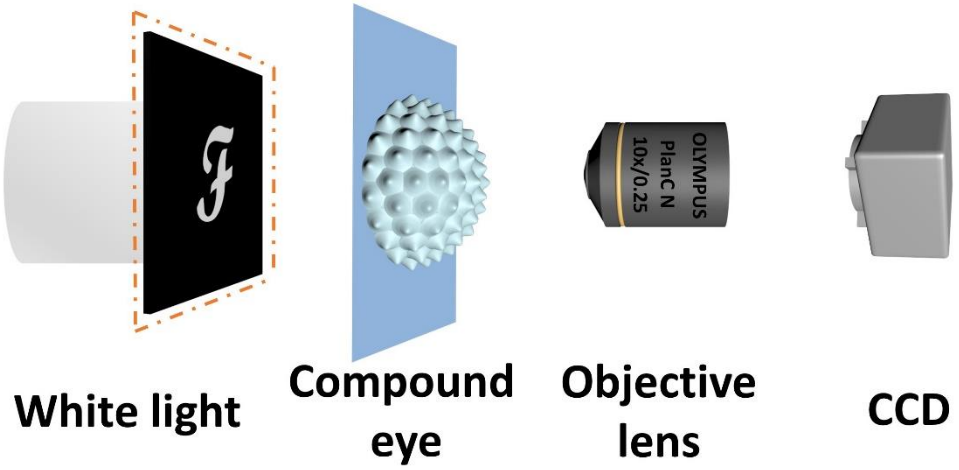

Supplementary Figure 4 | Schematic diagram of focusing and imaging test of μ-CEs. An objective lens (OLYMPUS, 10x/0.25) and a charge-coupled device (CCD) are used for microscopic imaging.

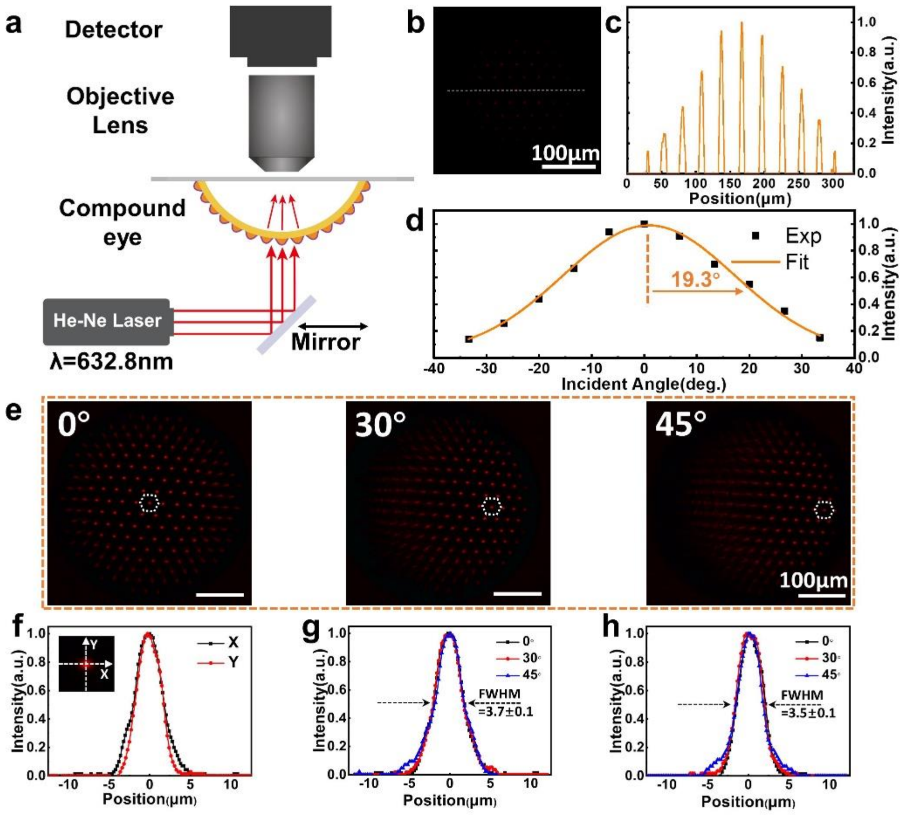

Supplementary Figure 5 | Measurement of FOV and ASF of the spherical μ-CE. (a) Schematic diagram of the experimental setup with an objective (OLYMPUS, 10x/0.25). Different angles of incidence are achieved by moving and rotating the mirror. **(b)** Under normal incidence, the optical photograph of the top ommatidium in focus. **(c)** The normalized intensity distribution extracted along the dotted line in (b). **(d)** ASF of the fabricated spherical μ-CE (FWHM=19.3°). **(e)** Focused light field images obtained by incident the spherical μ-CE at angles of 0°, 30°, and 45°, respectively. **(f)** Comparison of the intensity distributions along the x-axis and y-axis at normal incidence. Insert is an image of a focal point. Under different incident angles (0°, 30°, and 45°), the normalized intensity distribution along the X direction **(g)** and Y direction **(h)**. The wavelength of the continuous light used to test is 633nm.

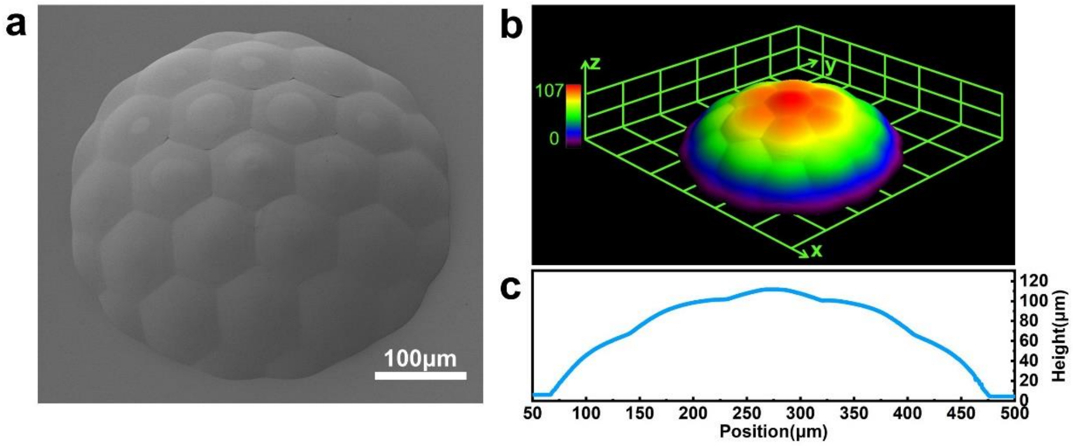

Supplementary Figure 6 | Morphological characterization of the logarithmic μ-CE with photoelectric integration for 3D reconstruction. (a) SEM picture (30° top view). **(b)** 3D LSCM picture. **(c)** Section profile curve.

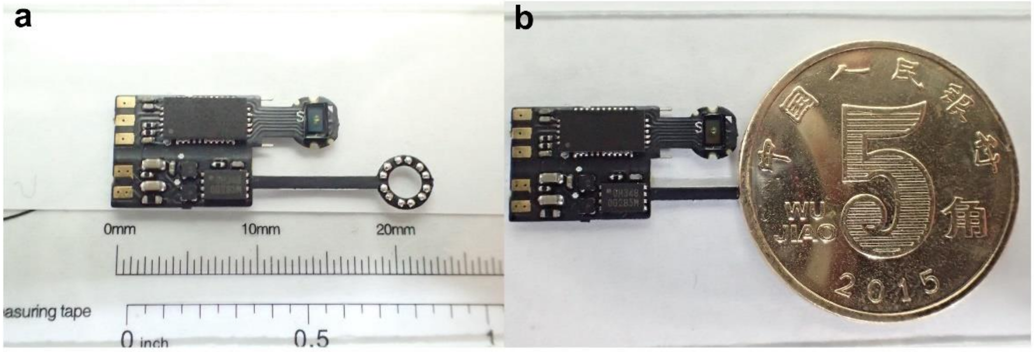

Supplementary Figure 7 | Photos of μ-CE camera modules (including μ-CE, CMOS chip, and driving circuit) with different reference objects. Reference object: **(a)** A ruler with scales. **(b)** A 5-dime coin in RMB.

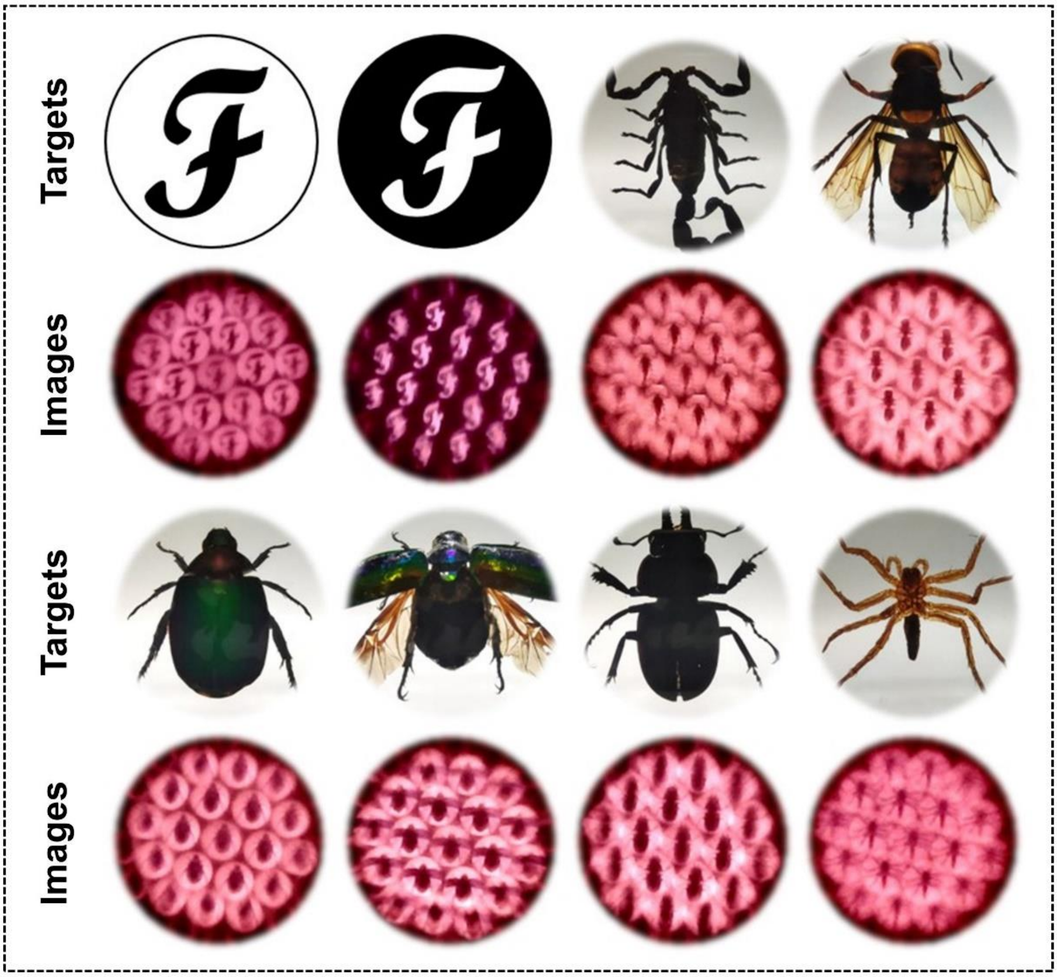

Supplementary Figure 8 | Photographs of different targets (top) and the images collected by the μ-CE camera (bottom). Magnified display of Figure 4a in the manuscript.

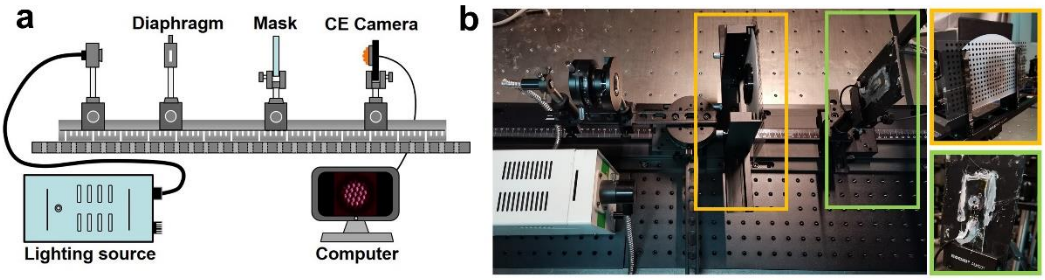

Supplementary Figure 9 | Schematic **(a)** and photos **(b)** of the experimental setup used for spatial calibration of the μ-CE camera.

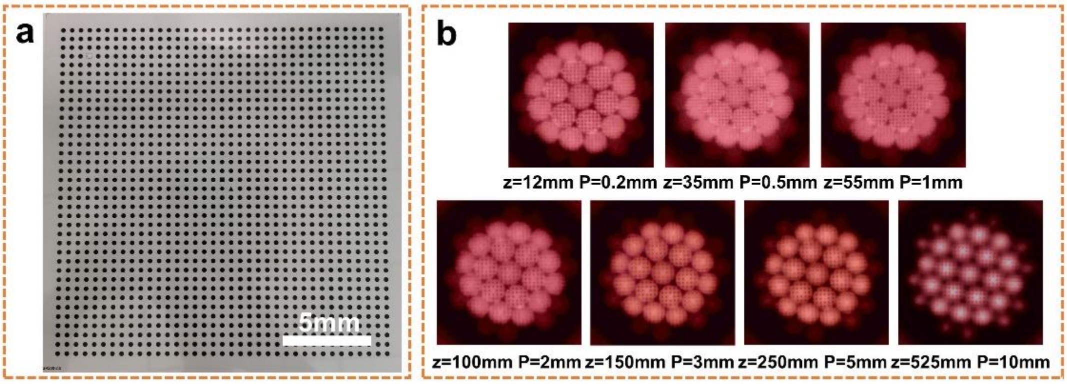

Supplementary Figure 10 | Macro calibration of the μ-CE camera. (a) The photo of the mask with dot pattern (period of 0.5mm) used for calibration. **(b)** Images of the mask with different dot periods collected by the μ-CE camera at different distances. Due to the large stroke, the slide rail is used to control the movement in steps of 1mm.

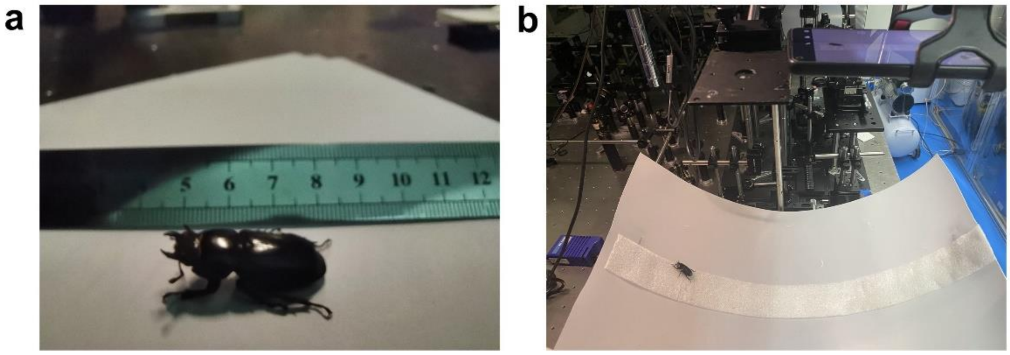

Supplementary Figure 11 | Reconstruction of the beetle's trajectory. (a) The photo of a beetle using a ruler as a reference. The length of the beetle is determined to be 30 mm. **(b)** The photo of the experimental setup used for 3D trajectory reconstruction. A smartphone and an μ-CE camera simultaneously collect beetle moving images.

Definition calculation for ommatidium imaging

In this experiment, the definition of ommatidium imaging was calculated using MATLAB platform based on the image definition evaluation function. The gray gradient function is a typical image definition evaluation function based on the spatial domain [1], which mainly includes: Energy of gradient function, Roberts gradient function, Sobel gradient function, Brenner gradient function, and Variance gradient function. Among them, the Brenner gradient function algorithm is also called the gradient filter method. It only needs to calculate the difference between the intensities of two pixels in the X direction, that is, the two-step gradient, which requires less calculation. The specific calculation expression is as follows:

$$D = \sum_{x} \sum_{y} \{[I(x+2, y) - I(x, y)]^2\}$$

Where D represents the calculated image definition, and $I(x, y)$ represents the gray value of the image pixel (x, y).

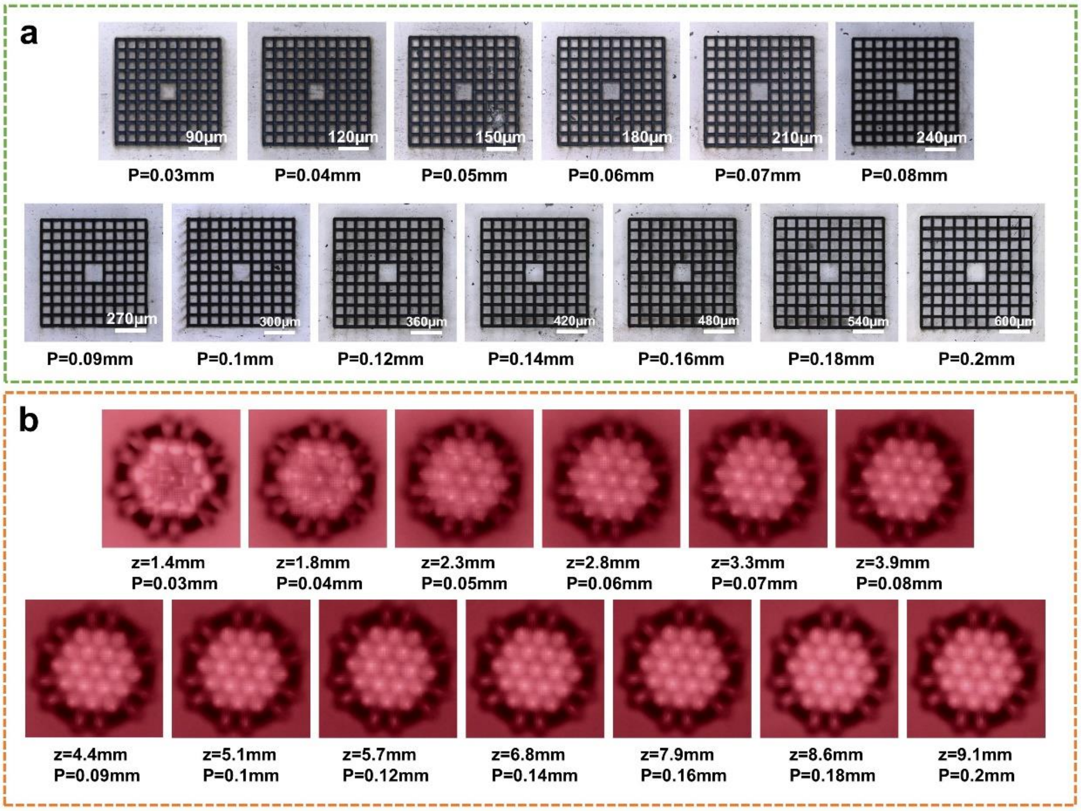

Supplementary Figure 12 | Micro calibration of the μ-CE camera. (a) The photo of the mask with a grid pattern (period of 0.03-0.2mm) used for calibration. The high-precision mask is made via ablating the glass surface with a high-energy femtosecond laser. **(b)** Mask images of different point periods collected by the μ-CE camera at different distances for spatial calibration. Due to the small stroke and high-accuracy requirements, a precision stage is used to control the movement in steps of 0.01 mm.

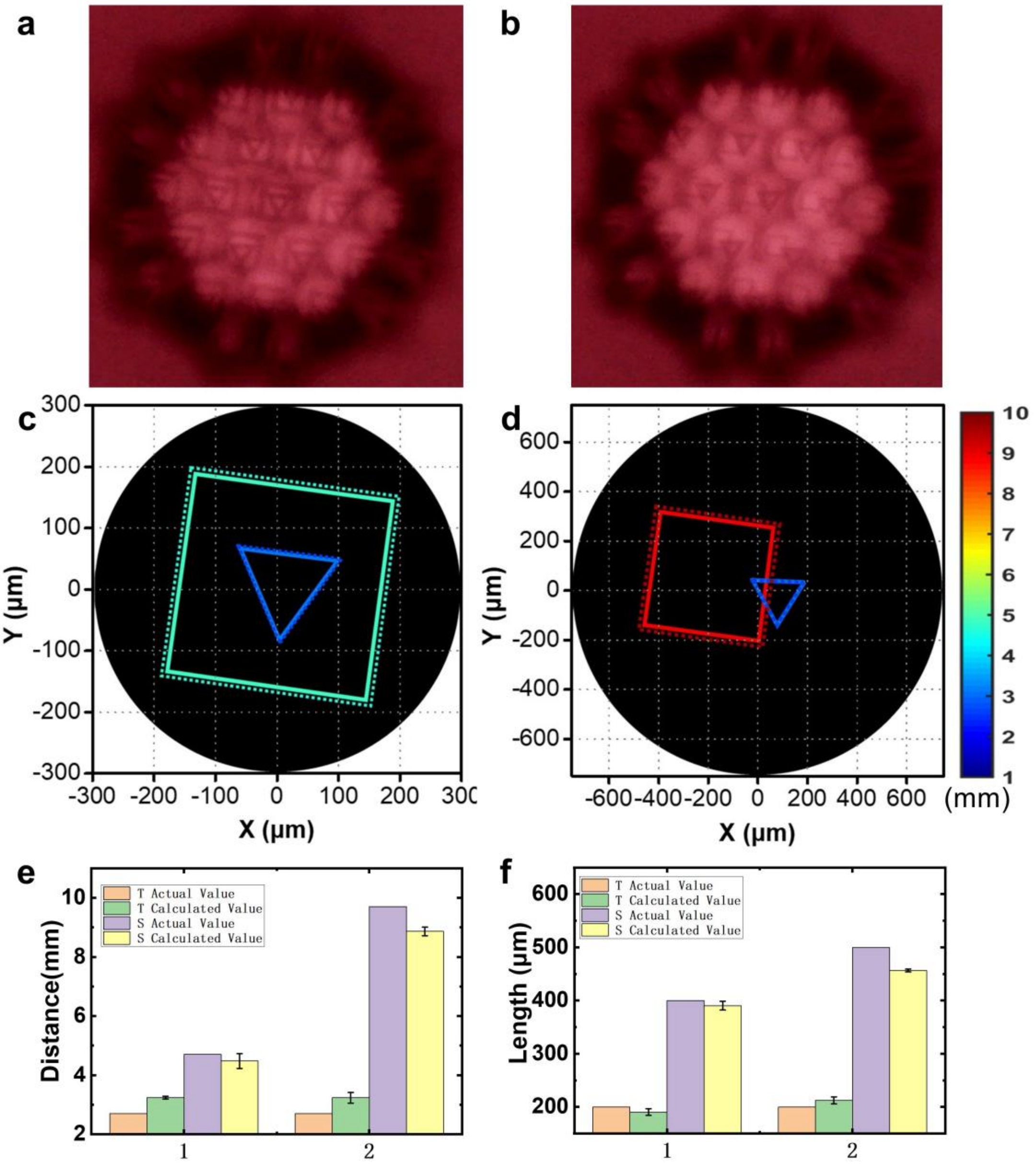

Supplementary Figure 13 | Quantitative statistics of micro-reconstruction results. (a, b) Unlabeled original images directly collected by the optoelectronic μ-CE camera. **(c, d)** Magnified 3D reconstruction results of the target objects correspond to **Figure 5(b) and (c)** in the manuscript. **(e)** Comparison of actual and calculated distances between triangle structure and square structure. **(f)** Comparison between actual and calculated side lengths of the triangle structure and the square structure. Among them, group 1 corresponds to **Figure 5(b)**, and group 2 corresponds to **Figure 5(c)**. Error bars denote the standard deviation of the measurements.

Supplementary Table 1. State-of-the-art of miniature compound eye imaging systems.

Defocus Problem& Solution	Integration type	Fabricating technology	100% fill factor	CE diameter	Ommatidium number	FOV	Image resolution	Ref(year)
Unsolved	no detector	Femtosecond laser two-photon polymerization	√	84μm	≈150	90°	—	[2] (2014)
Unsolved	no detector	Femtosecond laser wet etching and Thermal embossing	√	5mm	≈30000	120°	—	[3] (2016)
Unsolved	no detector	Femtosecond laser dry etching and Thermal embossing	√	1cm	>190000	90°	—	[4] (2019)
Cutting photodetectors	curved detector	Thermal reflow and UV molding	×	12.8mm	630	180°x60°	630	[5] (2013)
Hemispherical silicon photodiode array	curved detector	Casting and Curing	×	14.7mm	256	140°-180°	180	[6] (2013)
Optical relay system	planar detector	Soft lithography and Thermal embossing	×	40mm	4400	122.4°	1760000	[7] (2017)
Multi-layer Lens Assembly	no detector	Microinjection molding and Diamond machined	×	≈9mm	≈61	87°	—	[8] (2013)
Multi-layer Lens Assembly	planar detector	Femtosecond laser two-photon polymerization	×	≈2mm	5-28	170-180°	—	[9] (2022)
Curved multi-focus (theoretical)	no detector	Theoretical	×	870μm	121	60°	—	[10] (2010)
Curved multi-focus	no detector	Electrostatic deformed molding	×	≈700μm	37	36.6°	—	[11] (2014)
Curved multi-focus	planar detector	Hybrid imprinting	×	20mm	12	—	—	[12] (2017)
Logarithmic lens	planar detector	Femtosecond laser two-photon polymerization	√	400μm	19-160	90°	>80000	this work

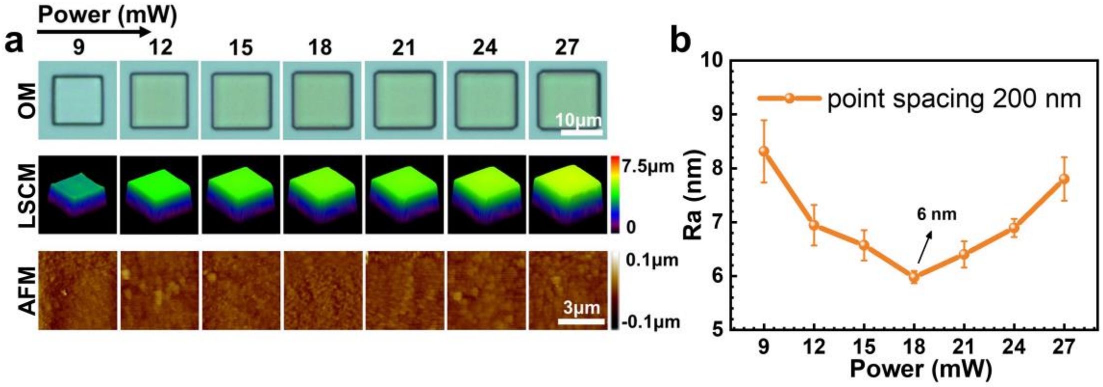

Supplementary Figure 14 | Surface smoothness optimization. (a) Optical microscope (OM), laser scanning confocal microscope (LSCM), and atomic force microscope (AFM) images of microcubes fabricated via various laser power. (b) Dependence of surface roughness on laser power. Considering the fabrication efficiency, voxel spacing (200 nm) and single-point exposure time (300 μs) are fixed. Error bars denote the standard deviation of the measurements.

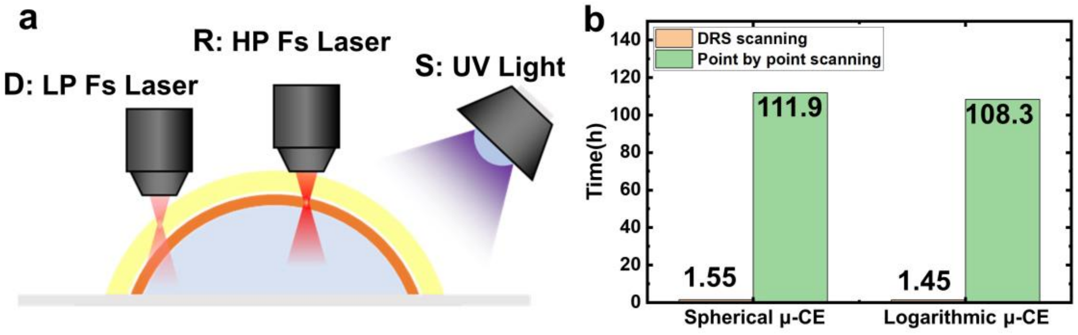

Supplementary Figure 15 | Comparison between the traditional point-by-point scanning strategy and the proposed DRS-TPP strategy. (a) Schematic diagram of DRS-TPP processing principle. D (definition): use a low-power femtosecond laser to accurately define the outer contour of the structure (scanning three layers). R (reinforcement): use a high-power femtosecond laser to perform enhanced scanning to improve the mechanical stability of the structure (scanning two layers); S (solidification): use ultraviolet light to quickly expose unpolymerized resin inside the structure. **(b)** Comparison of the processing time of spherical and logarithmic μ-CEs (diameter: 400 μm, height: 90 μm) prepared using the point-by-point scanning strategy and the DRS strategy. The spacing of the 3D point cloud data is 200 nm.

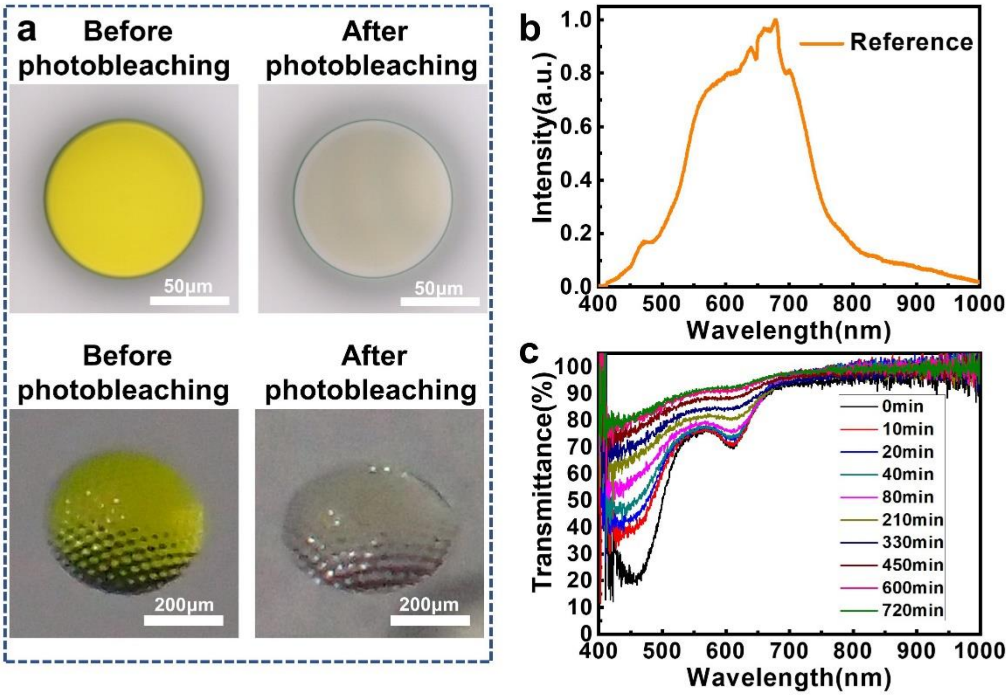

Supplementary Figure 16 | Optical transmittance of prepared microstructures in the visible wavelength range. (a) Optical photographs of the prepared micro cylinder (100μm in diameter, 50μm in height) and μ-CE (400μm in diameter, 90μm in height) before and after photobleaching for 12 hours. **(b)** The emission spectrum of the white light used for the test. **(c)** The transmission spectrum (400-1000nm) changes of the prepared microcylinder during photobleaching. A home-made micro-area transmission spectrum test system was used. After bleaching for 12 hours, the transmittance of the device reaches 93% at a wavelength of 633 nm.

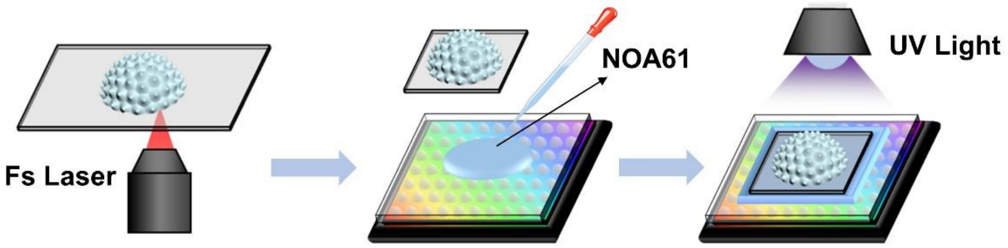

Supplementary Figure 17 | Integrated scheme of prepared μ-CEs and CMOS photosensitive chips. First, the μ-CE is prepared on the glass surface by FL-TPP, then NOA61 is dropped on the CMOS chip and the cut ACE is placed, and finally, the package is completed by UV exposure curing.

Supplementary References

[1] Mo, Chunhong, and Bo Liu. "An auto-focus algorithm based on maximum gradient and threshold." 2012 5th International Congress on Image and Signal Processing. IEEE, 2012.
[2] Wu D, et al. Bioinspired fabrication of high‐quality 3D artificial compound eyes by voxel‐modulation femtosecond laser writing for distortion‐free wide‐field‐of‐view imaging. Adv. Opt. Mater. 2, 751-758 (2014).
[3] Deng Z, et al. Dragonfly‐eye‐inspired artificial compound eyes with sophisticated imaging. Adv. Funct. Mater. 26, 1995-2001 (2016)
[4] Liu X Q, et al. Rapid engraving of artificial compound eyes from curved sapphire substrate. Adv. Funct. Mater., 29, 1900037 (2019).
[5] Floreano, D. et al. Miniature curved artificial compound eyes. PNAS 110, 9267-9272 (2013).
[6] Song, Y. M. et al. Digital cameras with designs inspired by the arthropod eye. Nature 497, 95-99 (2013).
[7] Shi C, et al. SCECam: a spherical compound eye camera for fast location and recognition of objects at a large field of view. Opt. Express, 25, 32333-32345 (2017).
[8] Zhang H, et al. Development of a low cost high precision three-layer 3D artificial compound eye. Opt. Express, 21, 22232-22245 (2013).
[9] Toulouse A, et al. Ultra-compact 3D-printed wide-angle cameras realized by multi-aperture freeform optical design. Opt. Express, 30, 707-720 (2022).
[10] Zhang Y, et al. Artificial compound-eye imaging system with a large field of view based on a convex solid substrate, Proc. SPIE 7848, 78480U (2010).
[11] Sun H, et al. Fabrication of microlens arrays with varied focal lengths on curved surfaces using an electrostatic deformed template. J. Micromech. Microeng. 24, 065008 (2014).
[12] Chen J, et al. Hybrid imprinting process to fabricate a multi-layer compound eye for multispectral imaging. Opt. Express, 25, 4180-4189 (2017).
