# Supplementary material for: Miniature optoelectronic compound eye camera
Source: Nat Commun. 2022 Sep 26;13:5634. doi: 10.1038/s41467-022-33072-8 (PMC9513083; doi:10.1038/s41467-022-33072-8)
Supplement: Supplementary file 2 — Description of Additional Supplementary Files [file 41467_2022_33072_MOESM2_ESM.pdf]

Description of Additional Supplementary Files

File name: Supplementary Movie 1
Description: The motion of a living beetle was recorded via a traditional camera and a μ-CE camera. To shorten the playback time, videos of the motion process are played 3 times faster.

File name: Supplementary Movie 2
Description: A paramecium motion video was recorded by the μ-CE camera, and the real-time trajectory reconstruction is performed at a rate of 24 frames per second.
